# Nutritional strategies of British professional and amateur natural bodybuilders during competition preparation

**DOI:** 10.1186/s12970-019-0302-y

**Published:** 2019-08-22

**Authors:** A. J. Chappell, T. Simper, E. Helms

**Affiliations:** 1 School of Pharmacy and Life Science, Robert Gordon University, Garthdee Road, Aberdeen, AB10 7AQ UK; 2 Sheffield Hallam University, Howard Street, Sheffield, S1 1WB UK; 3 Sports Performance Research Institue New Zealand, Auckland University of Technology, 17 Antares Place, Rosedale, Auckland, 0632 New Zealand

**Keywords:** Natural, Bodybuilding, Drug free, Competition preparation, Dietary strategies, Nutrition, Physique contest, Supplementation, Dieting, Professional

## Abstract

**Background:**

To prepare for competition, bodybuilders employ strategies based around: energy restriction, resistance training, cardiovascular exercise, isometric “posing”, and supplementation. Cohorts of professional (PRO) natural bodybuilders offer insights into how these strategies are implemented by elite competitors, and are undocumented in the scientific literature.

**Methods:**

Forty-seven competitors (33 male (8 PRO, 25 amateur (AMA), 14 female (5 PRO, 9 AMA) participated in the study. All PROs were eligible to compete with the Drug Free Athletes Coalition (DFAC), and all AMAs were recruited from the British Natural Bodybuilding Federation (BNBF). Competitors in these organisations are subject to a polygraph and are drug tested in accordance with the World Anti-Doping Agency. We report the results of a cross-sectional study of drug free bodybuilders competing at BNBF qualifying events, and the DFAC and World Natural Bodybuilding Federation finals. Participants completed a 34-item questionnaire assessing dietary intake at three time points (start, middle and end) of competition preparation. Participants recorded their food intake over a 24-h period in grams and/or portions. Dietary intakes of PRO and AMA competitors were then compared. Repeated measures ANOVA was used to test if nutrient intake changed over time, and for associations with division.

**Results:**

Male PROs reported significantly (*p* < 0.05) more bodybuilding experience than AMAs (PRO: 12.3 +/− 9.2, AMA: 2.4 +/− 1.4 yrs). Male PROs lost less body mass per week (PRO: 0.5 +/− 0.1, AMA: 0.7 +/− 0.2%, *p* < 0.05), and reported more weeks dieting (PRO: 28.1 +/− 8.1, AMA: 21.0 +/− 9.4 wks, *P* = 0.06). Significant differences (*p* < 0.05) of carbohydrate and energy were also recorded, as well as a difference (*p* = 0.03) in the estimated energy deficit (EED), between male PRO (2.0 +/− 5.5 kcal) and AMA (− 3.4 +/− 5.5 kcal) competitors.

**Conclusions:**

Longer diets and slower weight loss utilized by PROs likely contributed towards a lower EED compared to the AMAs. Slower weight loss may constitute an effective strategy for maintaining energy availability and muscle mass during an energy deficit. These findings require corroboration, but will interest bodybuilders and coaches.

**Electronic supplementary material:**

The online version of this article (10.1186/s12970-019-0302-y) contains supplementary material, which is available to authorized users.

## Introduction

In competitive bodybuilding athletes are judged on their aesthetics e.g. muscle size, proportions, and appearance of low body fat [1]. Bodybuilders train for years to build lean body mass (LBM), then follow meticulous pre-competition regimes for months to reduce body fat to showcase their physiques [2–9]. To prepare for competitions, athletes employ year round nutrition and training strategies based on an on-season, “competition preparation phase” and an off-season, “recovery/bulking phase” [10]. In addition to regular resistance training, during contest preparation the majority of bodybuilders follow high protein, calorie-restricted diets, aerobic exercise, and isometric “posing practice” to prepare for the mandatory physique poses which judges use to place competitors [11–13]. As the competition approaches, bodybuilders tend to increase physical activity and employ greater degrees of energy restriction [13]. Aside from losing body fat, a main aim of competition preparation is preventing the loss of LBM associated with energy deficits and low energy availability (EA) [14–16]. For example, one amateur (AMA) bodybuilder whose body mass losses during competition preparation consisted of over 40% LBM [17, 18], whereas in non-drug tested bodybuilding, such losses may be mitigated by anabolic steroids [19–22]. Thus, strategies to preserve LBM are a priority in “natural”, or drug free bodybuilding.

Recently, a cross-sectional study of high level British natural bodybuilders revealed that higher placing bodybuilders followed high protein (3.3 g/kg BW), low fat (0.6 g/kg BW) diets and consumed more carbohydrate and energy than their less successful peers [13]. These findings along with previous research further refine the evidence-based nutritional recommendations for natural bodybuilding contest preparation, by providing real world context for how dietary strategies affects bodybuilding performance [11, 23]. There remains, however, a paucity of research on bodybuilders. Most research is non-specific regarding the drug free status of the cohort and is restricted to small cross-sections or case studies. Of the former, most cross-sections were carried out in the late 80’s and early 90’s save for a few recent additions [24]. Furthermore, with the exception of Mitchell et al.’s [10] study of nine Australian natural bodybuilders and Maestu et al.’s [25] study of 14 Estonian bodybuilders, laboratory based observations have been carried out as case studies reporting the practices of American AMA bodybuilders who consume less energy than their British counterparts [5–9, 26–28].

One unexplored area is the nutritional strategies of “elite” professional (PRO) competitors. Professional athletes are regarded as elite examples of their sport. Moreover, natural bodybuilders are generally regarded to follow “evidenced based” approaches [29, 30], by comparison to those competing in the men’s physique category [31]. However, it is the observation of the authors - who are actively involved in competitive natural bodybuilding - that PRO status may not always reflect “elite”. Briefly, at any large enough amateur show, “PRO Cards” can be given to class winners, awarding them PRO status. PRO status therefore, does not guarantee one is competitive at the PRO level and distinguishing between elite and non-elite competitors is difficult. Qualitatively in the natural bodybuilding community, PROs placing in the top five of their classes at the Drug Free Athletes Coalition (DFAC) and World Natural Bodybuilding Federation (WNBF) PRO World Finals are regarded as elite. Nutritional strategies of these elite PROs therefore merit investigation. In the present investigation, we compared nutritional strategies of male and female British elite PRO and AMA bodybuilders preparing for competition. We sought to identify if there were differences in competition preparation strategies between PRO and AMA bodybuilders. This research will be of interest to coaches and competitive bodybuilders seeking to understand the nutritional principles and practices important to bodybuilding success. Furthermore, this research will also interested those wishing to maintain muscle mass while maintaining an energy deficit.

## Methods

### Design

All AMA and PRO male and female participants were recruited from British Natural Bodybuilding Federation (BNBF) regional qualifiers and the DFAC British PRO Grand Prix during 2017. One additional British Male PRO eligible to compete under the BNBF/DFAC natural criteria was recruited prior to participating in the WNBF World Championships. All competitors who won their class at BNBF regional qualifiers were subject to urine analysis drug testing, and the top three at the DFAC British PRO Grand Prix and WNBF World Championships were drug tested as well. All PRO competitors were subject to polygraph administered by a qualified polygrapher (to verify natural status). All DFAC PROs signed a waiver declaring their compliance with the World Anti-Doping Agency Code [32, 33]. A certified WADA laboratory (The Sports Medicine Research and Testing Laboratory, Salt Lake City, USA) carried out all testing on BNBF and DFAC samples.

The study was advertised via social media, and competitors were recruited in person by the first author (AC) at events. Participants were informed of study aims and methods via participant information sheets; those agreeing to participate provided written informed consent. This study was approved by the university ethics board. Participants then completed a 34-item questionnaire (see Additional file 1) on dietary habits and BW change at three time points: start, middle, and end phase of the competition diet. Participants retrospectively recorded their food intake over a 24-h period in grams and/or portions at bodybuilding events. Missing data, and/or clarification of portion sizes, weights, supplement brands etc. were followed up via email. Results are reported separately for both sexes, and PRO and AMA divisions. Males were from the bodybuilding class, while females were from the bodybuilding, athletic and figure classes. The athletic and figure class emphasise less muscularity compared to bodybuilding; body fat levels distinguish the two categories i.e. lower for athletic and higher for figure.

### Participant characteristics and estimated energy deficit

Competitors self-reported BW at the start (initial weight) and end (prior to taking part in the competition) of their contest preparation. Total weight loss, and percentage weight loss were calculated as the difference between the start and end. Body mass index (BMI) was calculated from self-reported height as kg / m^2^. Participant’s basal metabolic rate (BMR) was calculated using the Schofield equations [34]. The Schofield equations estimate BMR based on age, sex and bodyweight. A physical activity and lifestyle (PAL) factor of 1.7 (equating to a moderately active individual) [34] was used for all competitors and was multiplied by BMR to estimate daily energy requirements. Finally, the estimated energy deficit (EED) was calculated by subtracting BMR × PAL from total energy intake, scaled to body mass.

### Dietary analysis

Nutritional analysis was performed using Nutritics nutrition analysis software (version 5.092 Academic Edition, Nutritics, Dublin, Ireland). Total macronutrient, energy and caffeine intake was reported as grams (g), kilocalories (kcal) and milligrams (mg) per day, respectively. Macronutrients as g per kg of BW (g/kg BW), energy intake as kcal per kg of BW (kcal/kg BW) and caffeine as mg per kg of BW (mg/kg BW) were calculated for start and end, based on competitors’ reported bodyweight. Macronutrient and caffeine information from dietary supplements and beverages was derived from brand websites. The number of food items consumed at each phase of preparation was counted. The percentage of the diet made up of specific food groups was calculated based on the European Food Safety Agency food classification system for dietary reporting [35]. Any food group contributing to less than 1% of food group intake was placed in the other ingredients category. Beverages were reported separately. No competitor reported consuming sugar sweetened beverages or alcohol during their regular diet. Competitors’ fluid intake, and whether or not they consumed artificial sweeteners or sugar free cordials, was recorded as a binary variable.

### Supplements

Supplements were split into 15 categories based on previous research [13] including: protein powder, branched chain amino acids (BCAA), vitamin C, omega 3 fatty acids, multivitamins, creatine, vitamin D, pre-workout supplements, carbohydrate (CHO) powders, individual amino acids, fat burners, mineral supplements, joint supplements, protein bars and miscellaneous supplements (supplements used too infrequently to be categorised). The number of supplements used by PRO and AMA competitors was reported as a percentage of their usage by the cohort.

### Statistical analysis

Analysis was performed using IBM Statistical Package for the Social Sciences (version 25). Normality was assessed using the Shapiro-Wilks test, where data was not normally distributed the Wilcoxon signed rank test was implemented and results expressed as medians and IQR. Comparisons between male and female PROs and AMAs was carried out with repeated measures analysis of variance (ANOVA). The effect of time, division, and time × division was examined. Mauchly’s test of sphericity was applied to data and where this was violated the Greenhouse-Geisser estimate was utilized. Independent T-Tests were used to identify if there was a difference in means between PRO and AMA relating to: i) age, ii) years bodybuilding and competing, iii) height, iv) diet duration, v) diet start and end weight, vi) total weight loss, vii) weight loss per week, ix) % weight loss, x) % weight loss per week, xi) start and end BMI, xii) start and end EED, xiii) supplement usage, xiv) fluid intake and xv) food selection patterns. Categorical variables were analysed using the Pearson Chi-squared test for: i) artificial sweetener intake, ii) sugar free fruit cordial intake, and iii) beverage intake. Statistical significance was set at *p* < 0.05. Pooled standard deviations were used to calculate Cohen’s *d,* and effect sizes multiplied by 0.975, to correct for bias and produce *d*. Effect size cutoffs and confidence intervals (CI) were based on Hopkins suggestions for sports science: < 0.2, 0.2–0.6, 0.6–1.2, 1.2–2.0, and 2.0–4.0, for trivial, small, moderate, large, and very large effects. Data are presented as means and standard deviations unless otherwise stated.

## Results

### Participant characteristics

Forty seven natural bodybuilders (33 male) were recruited. The male cohort included 8 PROs and 25 AMAs. All male PROs had placed in the top five of DFAC or WNBF PRO World Finals. The cohort also included 3 competitors who had won their weight class at the aforementioned World Finals, and a two-time overall PRO World champion. The female cohort included 14 competitors: 5 PROs (4 figure, 1 bodybuilding), and 9 AMAs (5 figure, 2 masters figure, 1 bodybuilding, 1 athletic). Amongst the PRO females, 3 previously placed in the top 3 at the DFAC PRO World Finals. Complete dietary information was available for all participants. Participant characteristics including diet length are presented in Table 1.

**Table 1:** Self-reported Characteristics of British Professional and Amateur Natural Bodybuilders Preparing for Competition

	Male competitors					Female competitors					Male		Female	
	PRO	SD	AMA	SD	*P* value	PRO	SD	AMA	SD	*P* value	Mean	SD	Mean	SD
Age	34.9	8.6	29.7	8.5	0.147	45.6	9.3	36.0	7.9	0.064	30.9	8.7	39.4	9.4
Years Bodybuilding	17.0	8.4	11.2	7.8	0.083	4.3	2.3	5.3	2.7	0.667	12.6	8.2	4.9	3.8
Years Competing	12.3	9.2	2.4	1.4	> 0.001	3.6	2.4	2.7	1.2	0.348	4.8	6.2	3.0	1.7
Height (m)	1.77	0.05	1.77	0.1	0.865	1.64	0.1	1.62	0.1	0.527	1.77	0.05	1.63	0.1
Diet Length (weeks)	28.1	8.1	21.0	9.4	0.064	25.0	7.3	25.0	8.3	1.000	22.7	9.5	25.0	7.7
Diet Start Weight (kg)	95.6	8.3	88.3	8.0	0.032	65.5	5.9	65.7	6.0	0.964	90.1	8.5	65.6	5.8
Diet End Weight (kg)	82.3	6.6	74.9	6.6	0.010	57.4	4.7	56.0	4.7	0.597	76.7	7.2	56.5	4.6
Total Weight Loss (kg)	13.4	6.1	13.1	5.2	0.916	8.1	1.6	9.7	3.3	0.336	13.2	5.4	9.1	2.8
Weight Loss Per Week (kg)	0.5	0.2	0.6	0.2	0.936	0.3	0.1	0.4	0.1	0.367	0.5	0.2	0.3	0.1
% Weight Loss	13.8	5.5	14.7	5.4	0.681	12.3	1.7	14.6	4.2	0.272	14.5	5.3	13.8	3.6
% weight loss per week	0.5	0.1	0.7	0.2	0.008	0.5	0.1	0.6	0.2	0.336	0.7	0.2	0.6	0.2
Start BMI (kg/m2)	30.4	1.5	28.4	2.5	0.038	24.2	1.4	25.0	2.1	0.446	28.9	2.5	24.7	1.9
End BMI (kg/m2)	26.2	1.01	24.0	1.7	> 0.001	21.2	1.2	21.3	1.2	0.915	24.5	1.8	21.3	1.2

*p* value, difference in means between Pros and Amateur competitors, Male competitors Pros n - 8, Amateur n - 25, Male Mean n - 33, Female competitors PRO n – 5, AMA n – 9, Female Mean n - 14. Data analysed using a student t test where *p* < 0.05 equals statistical significance

*Abbreviations*: *PRO* professional, *AMA* amateur, *SD* standard deviation, *BMI* body mass index

### Nutrient intake

#### Energy and macronutrients intake

Total macronutrient and energy intake for the start, middle and end of contest preparation are reported in Table 2. Results of the repeated measures ANOVA identified a significant reduction in energy and macronutrients as preparation progressed (*p* time < 0.05) in both males and females. Total CHO and energy intake was significantly higher (*p* division < 0.05) in PRO men compared to AMAs. Furthermore, there was a trend for a higher fibre intake in PRO men compared to AMAs (*p* division = 0.068) as well as an interaction over time (*p* time x division = 0.079). Cohen’s *d* effect size analysis indicated a large effect size for a higher total CHO (start: *d* = 1.1 CI [0.3, 2.0], middle: *d* = 1.1 CI [0.3, 2.0], end: *d* = 1.4 CI [0.5, 2.4]) and energy intake (start: *d* = 1.1 CI [0.3, 2.0], middle: *d* = 0.9 CI [0.1, 1.8], end: *d* = 1.4 CI [0.6, 2.3]) in PRO men compared to AMAs. No other significant effect sizes were detected between PRO and AMA men: protein start *d* = 0.5 CI [0.0, 1.3], middle *d* = 0.4 CI [− 0.4, 1.2], end *d* = 0.5 CI [− 0.3, 1.3]; fat start *d* = − 0.2 CI [− 1.0, 0.6], middle *d* = − 0.5 CI [− 1.3, 0.3], end *d* = 0.1 CI [− 0.9, 0.7]; fibre start *d* = 0.8 CI [0.0, 1.7], middle *d* = 0.5 CI [− 0.3, 1.3], end *d* = 0.8 CI [0.0, 1.7]. Among females no significant effect sizes were detected between PROs and AMAs: protein start *d* = 0.0 CI [− 1.1, 1.1], middle *d* = 0.1 CI [− 1.0, 1.2], end *d* = 0.1 CI [− 1.0, 1.2]; CHO start *d* = 1.1 CI [− 0.4, 1.9], middle *d* = 0.2 CI [− 0.4, 1.9], end d = 0.5 CI [− 0.6, 1.6]; fat start *d* = 0.2 CI [− 1.3, 0.9], middle *d* = 0.5 CI [− 0.6, 1.7], end *d* = 0.0 CI [− 1.1, 1.1]; fibre start *d* = 0.3 CI [− 0.8, 1.5], middle *d* = 0.1 CI [− 1.7, 1.1], end *d* = 0.3 CI [− 0.8. 1.4]; energy start *d* = 0.4 CI [− 0.7, 1.5], middle *d* = 1.0 CI [− 0.2, 2.2], end *d* = 0.4 CI [− 0.7, 1.6]. As a percentage of energy intake, macronutrient intake among males was: CHO PRO 49.2 to 49.7%, AMA 39.8 to 43.4%; protein PRO 31.2 to 34.0%, AMA 34.0 to 39.3%; fat PRO 13.2 to 15.3%, AMA 17.7 to 19.7%. Macronutrients as a percentage of energy among females was: CHO PRO 36.8 to 44.1%, AMA 31.5 to 39.0%; protein PRO 34.6 to 43.0%, AMA 36.7 to 45.5%; fat PRO 17.8 to 22.5%, AMA 20.8 to 21.1%.

**Table 2:** Total Macronutrient and Energy of British Professional and Amateur Natural Bodybuilders

	Phase	PRO	SD	AMA	SD	Mean	SD	*p* time	*p* division	*p* time × division
Male										
Protein (g)	Start	276.7	82.1	247.8	45.3	254.8	56.3	*0.001*	*0.221*	*0.848*
	Middle	257.3	71.5	232.9	49.5	238.8	55.4			
	End	250.7	48.7	222.6	56.7	229.4	55.5			
Carbohydrate (g)	Start	461.3	100.6	346.9	97.7	374.6	108.9	*0.001*	*0.003*	*0.167*
	Middle	401.4	112.9	294.0	32.3	320.1	102.0			
	End	406.8	122.8	254.4	106.8	291.3	127.5			
Fat (g)	Start	60.8	26.4	65.5	25.7	64.3	25.5	*0.001*	*0.480*	*0.420*
	Middle	45.7	16.3	55.7	21.6	53.3	20.7			
	End	43.3	11.8	45.9	19.4	45.3	17.7			
Fibre (g)	Start	52.0	18.5	39.2	14.0	42.4	16.0	*0.001*	*0.068*	*0.079*
	Middle	41.6	17.1	35.3	12.1	36.9	13.5			
	End	45.1	17.5	32.4	14.1	35.6	15.8			
Energy (kcal)	Start	3533.1	528.6	2968.5	488.8	3105.4	548.4	*0.001*	*0.004*	*0.170*
	Middle	3053.2	587.2	2607.0	441.2	2715.1	509.0			
	End	3018.4	567.1	2329.7	436.7	2496.6	550.7			
Female										
Protein (g)	Start	209.4	38.4	209.3	40.9	209.4	38.4	*0.017*	*0.855*	*0.856*
	Middle	207.4	43.2	200.8	50.0	203.1	46.1			
	End	192.8	22.9	186.8	45.7	188.9	38.1			
Carbohydrate (g)	Start	288.8	68.3	237.9	66.8	256.1	70.4	*0.001*	*0.178*	*0.851*
	Middle	247.6	26.8	197.0	76.6	215.1	66.8			
	End	181.8	57.3	143.3	88.5	157.1	78.6			
Fat (g)	Start	52.1	25.1	56.5	27.2	54.9	25.6	*0.020*	*0.775*	*0.318*
	Middle	61.4	29.7	47.2	21.5	52.2	24.6			
	End	37.5	18.5	37.6	19.5	37.6	18.4			
Fibre (g)	Start	23.2	9.0	20.8	7.5	21.7	7.8	*0.002*	*0.668*	*0.740*
	Middle	27.2	4.2	27.5	6.8	27.4	5.7			
	End	30.6	5.9	28.6	5.6	29.4	5.5			
Energy (Kcal)	Start	2463.0	523.0	2299.6	347.2	2357.9	406.2	*0.001*	*0.166*	*0.601*
	Middle	2373.4	366.6	2016.5	334.6	2144.0	376.5			
	End	1835.8	301.9	1660.8	415.5	1723.3	376.6			

p time, difference in means over the competition preparation diet (start, middle and end of phases), p result, division in means between PRO and AMA. Time x division interaction between diet over time and division. Differences in macronutrients and energy measured by repeated measures ANOVA. Statistical significance assumed where *p* < 0.05

*Abbreviations*: Phase stage of the competition preparation, *PRO* professional, *AMA* amateur, *SD* standard deviation

Mean macronutrient and energy intake scaled for body mass is reported in Table 3. Repeated measures ANOVA identified a number of significant differences and trends for a reduction in fat (*p* time = 0.024), protein (*p* time = 0.060) and energy (*p* time = 0.089) during preparation among males. Male PROs also consumed significantly (*p* division = 0.034) more CHO than AMAs although not consistently over time (*p* time x division = 0.135). Effect size analysis indicated a large effect for greater CHO intake scaled to body mass among male PROs compared to AMAs (start *d* = 0.7 CI [− 0.2, 1.5], end *d* = 1.0 CI [0.2, 1.8]). No other significant effect sizes were detected between the male divisions: protein start *d* = 0.1 CI [− 0.7, 0.9], end *d* = 0.1 CI [− 0.7, 0.9]; fat start *d* = − 0.5 CI [− 1.3, 0.3], end *d* = − 0.2 CI [− 1.0, 0.6]; energy start *d* = 0.4 CI [− 0.4, 1.2], end *d* = − 0.8 CI [0.0, 1.6]. No significant effect sizes were detected between female PROs and AMAs: protein start *d* = 0.0 CI [− 1.1, 1.1], end *d* = 0.0 CI [− 1.1, 1.1]; CHO start *d* = 0.8 CI [− 0.3, 1.9], end *d* = 0.4 CI [− 0.7, 1.5]; fat start *d* = 0.1 CI [− 1.0, 1.2], end *d* = 0.1 CI [− 1.2, 1.0]; energy start *d* = 0.6 CI [− 0.5, 1.8], end *d* = 0.3 CI [− 0.8, 1.4].

**Table 3:** Macronutrient and Energy Intake Scaled for Body Size of British Professional and Amateur Natural Bodybuilders

	Stage	PRO	SD	AMA	SD	Mean	SD	*p* time	*p* division	*p* time × division
Male										
Protein (g/kg BW)	Start	2.9	0.9	2.8	0.6	2.9	0.7	*0.060*	*0.814*	*0.889*
	End	3.1	0.6	3.0	0.8	3.0	0.7			
Carbohydrate (g/kg BW)	Start	4.9	1.1	4.1	1.2	4.3	1.2	*0.228*	*0.034*	*0.135*
	End	4.9	1.3	3.5	1.5	3.8	1.8			
Fat (g/kg BW)	Start	0.6	0.3	0.8	0.3	0.8	0.3	*0.024*	*0.549*	*0.363*
	End	0.8	0.3	0.8	0.4	0.8	0.3			
Energy (kcal/kg BW)	Start	37.2	7.5	34.5	6.1	35.2	6.5	*0.089*	*0.124*	*0.301*
	End	36.6	5.4	31.4	6.6	32.6	6.4			
Female										
Protein (g/kg BW)	Start	3.2	0.6	3.2	0.8	3.2	0.8	*0.113*	*0.967*	*0.879*
	End	3.1	0.8	2.6	1.6	2.8	1.4			
Carbohydrate (g/kg BW)	Start	4.0	1.4	3.0	1.1	3.4	1.2	*0.152*	*0.201*	*0.626*
	End	3.1	0.8	2.6	1.6	2.8	1.4			
Fat (g/kg BW)	Start	0.8	0.5	0.7	0.3	0.7	0.4	*0.572*	*0.998*	*0.714*
	End	0.7	0.3	0.7	0.4	0.7	0.3			
Energy (kcal/kg BW)	Start	34.5	12.3	29.1	5.5	31.0	8.5	*0.827*	*0.251*	*0.554*
	End	32.2	3.1	30.2	7.6	30.9	6.3			

p time, difference in means over the competition preparation diet (start, middle and end of phases), p result, division in means between PRO and AMA. Time x division interaction between diet over time and division. Differences in macronutrients and energy measured by repeated measures ANOVA. Statistical significance assumed where *p* < 0.05

*Abbreviations*: Phase stage of the competition preparation, *PRO* professional, *AMA* amateur, *SD* standard deviation

#### Diet diversity

**Fig. 1:**
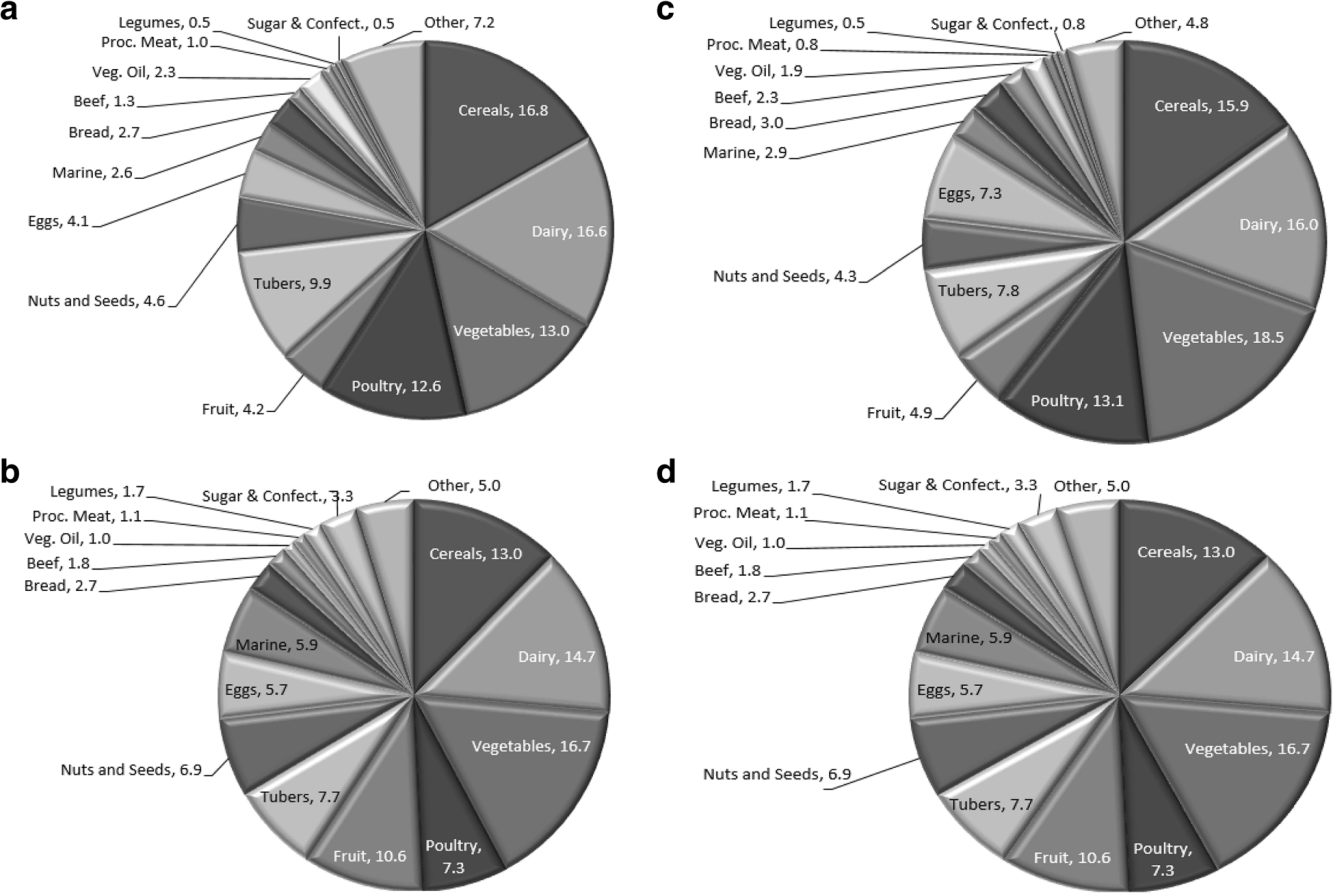
a–d Percentage Food Group Intake of British Natural Bodybuilders during Competition Preparation. **a** Professional Male Bodybuilders, **b** Amateur Male Bodybuilders, **c** Professional Female Bodybuilders, **d** Amateur Female Bodybuilders. Dairy products includes whey and casein supplements, Processed meats include, sausages, bacon, pies meat pastries etc., Fruit includes fruit products, Eggs includes egg products including egg protein isolate, Marine, includes fish, seafood, amphibians reptiles and invertebrates. Sugar includes confectionary, water- based sweet desserts and CHO powders e.g. glucose, dextrose, highly branched cluster dextrin. Other includes: all foods that fail to fit into the aforementioned categories. Abbreviations: Veg. vegetable, Proc. processed, CHO carbohydrate, Confect. Confectionary

Male PROs and AMAs reported 14.9 ± 4.9 and 15.8 ± 4.6 food items, while female PROs and AMAs reported 13.5 ± 4.4 and 16.7 ± 4.7 food items respectively, throughout preparation. There was no significant difference (Males: *t* (97) = 1.039, *p* = 0.303, females: *t* (40) = 1.044, *p* = 0.301) in the number of food items consumed throughout preparation between PROs and AMAs of either sex. The contribution different food groups make to PRO and AMA competitors’ diets is presented in Fig. 1a–d. Male PROs consumed more red meat (*z* = 2.326, *p* = 0.020), fruit (*z* = 2.206, *p* = 0.027), and sugar and confectionary items (*z* = 4.357, *p* < 0.001) than AMA. In contrast, male AMA consumed more, cereals (*z* = 2.398, *p* = 0.016), and eggs (*z* = 3.358, *p* = 0.001), than PROs. In the female cohort, AMAs consumed significantly more (*z* = 3.073, *p* = 0.002) poultry than PROs, while PROs consumed significantly more (*z* = 2.128, *p* = 0.033) food from marine sources than AMAs. No other significant differences (*p* >  0.05) were detected between sexes. Cereals, dairy, white meat and vegetables were the most popular food items consumed. Cereals were consumed mainly as oats and white or brown rice; dairy was consumed mainly as protein powder and yoghurt; white meat as poultry; and vegetables as broccoli, spinach and mushrooms. Other popular groups included tubers as white and sweet potatoes, fruit as raspberries and blueberries. No competitors reported consuming alcohol, sugar sweetened beverages, composite diet dishes, animal fats for cooking or food imitates e.g. Quorn.

### Estimated energy requirements and energy deficit

There was no significant difference (*p* >  0.05) between PRO and AMA, of either sex for estimated BMRs with and without the addition of PAL (Additional file 2). The EED was significantly greater in male AMA compared to PROs at the start (t (30) 2.57, *p* = 0.034, *d* = 1.0, CI [0.2, 1.9], PRO: 2.0 ± 5.5 kcal/kg BW, AMA: − 3.39 ± 5.2 kcal/kg BW) and end of preparation (t(31) 3.32, *p* = 0.002, *d* = 1.3, CI [0.5, 2.2], PRO: − 1.1 ± 6.0 kcal/kg BW, AMA: − 9.3 ± 6.1 kcal/kg BW). There were no significant differences or effect sizes detected for EED in the female cohort at either the start (t(12) 0.60, *p* = 0.558, *d* = 0.3 CI [− 0.7, 1.4], PRO: 1.8 ± 8.5 kcal/kg BW, AMA: − 0.8 ± 7.1 kcal/kg BW), or end (t(12) 0.49, *p* = 0.634, d = 0.3 CI [− 0.8, 1.4], PRO: − 7.3 ± 4.5 kcal/kg BW, AMA: − 10.0 ± 7.5 kcal/kg BW) of preparation.

### Dietary supplements

The number of supplements reported by male and female competitors was 6.7 ± 2.7, and 8.8 ± 1.8, respectively. There was a trend (t (31) = 1.71, *p* = 0.097) for male PROs to use more supplements (PRO: 8.1 ± 2.8, AMA: 6.3 ± 2.6). Dietary supplements reported by competitors are presented in Table 4. Moreover, female PRO consumed significantly more (t (12) = 2.54, *p* = 0.026) supplements than AMAs (PRO: 10.0 ± 1.6, AMA: 8.0 ± 1.5). The most frequently consumed supplements included: protein powders, branch chain amino acids, vitamin C, omega 3 fatty acids, multivitamins and creatine (Table 4). Miscellaneous supplements included: iron tablets, chromium, tribulus, medium chain triglycerides, green tea extract, kelp powder, digestive enzymes, and L-carnitine. Protein and energy intakes from supplements as a percentage of total protein and energy consumed was 28.8 ± 15.7% and 16.3 ± 10.3%, and 22.4 ± 9.6% and 15.3 ± 6.0% for males and females PROS and AMAs, respectively.

**Table 4:** Self-reported Supplement Usage of British Natural Bodybuilders during Competition Preparation

Supplement	Male				Female				Mean Intake			
	PRO	SD	AMA	SD	PRO	SD	AMA	SD	Men	SD	Women	SD
Protein powder (%)	100.0	5.8	100.0	9.8	100.0	1.7	100.0	2.3	100.0	0.0	100.0	0.0
Branch chain amino acids (%)	87.5	7.6	60.0	10.3	100.0	1.7	88.9	4.8	66.7	0.5	92.9	3.9
Vitamin C (%)	50.0	5.4	60.0	9.2	100.0	1.7	88.9	4.8	57.6	0.5	92.9	3.9
Omega 3 (%)	50.0	6.2	56.0	7.8	80.0	4.7	100.0	2.3	54.5	0.5	92.9	4.0
Multivitamin (%)	50.0	6.2	60.0	11.6	100.0	1.7	77.8	5.6	57.6	0.5	85.7	4.5
Creatine (%)	87.5	7.0	52.0	7.6	60.0	4.9	77.8	5.7	60.6	0.5	71.4	5.7
Vitamin D (%)	50.0	6.2	32.0	7.4	80.0	4.9	66.7	6.4	36.4	0.5	71.4	5.7
Pre-workouts (%)	62.5	7.2	64.0	10.7	40.0	5.6	22.2	4.5	63.6	0.5	28.6	4.8
Carb supplements (%)	87.5	5.7	24.0	5.7	60.0	4.9	11.1	4.8	39.4	0.5	28.6	5.0
Individual amino acid (%)	25.0	4.0	20.0	6.9	40.0	5.6	33.3	5.9	21.2	0.4	35.7	5.6
Fat Burners (%)	25.0	5.5	16.0	5.3	60.0	5.3	22.2	5.7	18.2	0.4	35.7	5.5
Joint supplement (%)	25.0	4.0	12.0	4.2	40.0	6.0	33.3	6.1	15.2	0.4	35.7	5.9
Mineral supplement (%)	37.5	5.7	12.0	3.4	40.0	6.5	22.2	6.5	18.2	0.4	28.6	6.3
Protein bars (%)	62.5	9.5	40.0	8.9	20.0	4.1	11.1	3.7	45.5	0.5	14.3	3.7
Miscellaneous supplements (%)	12.5	3.2	20.0	6.4	100.0	14.4	44.4	6.8	18.2	0.4	64.3	9.7

Values expressed as a percentage of the population who utilised a particular dietary supplement. Miscellaneous supplements, supplements that were used too infrequently to be designated as a category

*Abbreviations*: *PRO* professional, *AMA* amateur, *SD* Standard Deviation

### Caffeine, beverages, fluids and artificial sweeteners

There was no significant difference in reported caffeine intake (mg) in both sexes over time (male *p* = 0.732, female *p* = 0.467), between divisions (male *p* = 0.743, female *p* = 0.160), or interaction between time × division (male *p* = 0.558, female *p* = 0.423). Caffeine intake among males was: start PRO 236 ± 89 mg, AMA 210 ± 131 mg, mean 217 ± 120 mg; middle PRO 236 ± 89 mg, AMA 217 ± 130 mg, mean 222 ± 120 mg; end PRO 232 ± 83 mg, AMA end 229 ± 149 mg, mean end 230 ± 134 mg. Total caffeine intake among females was: start PRO 313 ± 67 mg, AMA 198 ± 98 mg, mean 233 ± 103 mg; middle PRO 313 ± 67 mg, AMA 212 ± 120 mg, mean 243 ± 114 mg; end PRO 313 ± 67 mg, AMA 237 ± 115 mg, mean 260 ± 106 mg. Caffeine intake scaled for body mass increased significantly over time in males (*p* = 0.021) and females (*p* = 0.026), however there was no difference between divisions (male *p* = 0.927, female *p* = 0.435), or interaction between time × division (male *p* = 0.407, female *p* = 0.204). Caffeine intake scaled for body mass among males was: start PRO 2.5 ± 1.1 mg/kg BW, AMA 2.4 ± 1.5 mg/kg BW, mean 2.4 ± 1.4 mg/kg BW; end PRO 2.9 ± 1.1 mg/kg BW, AMA 3.1 ± 2.1 mg/kg BW, mean 3.0 ± 1.9 mg/kg BW. Caffeine intake scaled for body mass among females was: start PRO 4.8 ± 1.2 mg/kg BW, AMA 3.1 ± 1.5 mg/kg BW, mean 3.6 ± 1.5 mg/kg BW; end PRO 5.5 ± 1.5 mg/kg BW, AMA 4.3 ± 2.1 mg/kg BW, mean 4.6 ± 1.9 mg/kg BW.

There was no significant difference (χ^2^ (1) 2.60, *p* = 0.11) between male PROs (87.5%) and AMAs (56.0%) in artificial sweetener usage. Among females there was a non-significant trend (χ^2^ (1) 3.75, *p* = 0.053) for AMAs (85.7%) to use sweeteners more than PROs (14.3%). There was no significant difference (χ^2^ (1) 0.083, *p* = 0.774) in sugar free cordial intake between male PROs (37.5%) and AMAs (32.0%). Furthermore, there was no significant difference in (χ^2^ (1) 0.44, *p* = 0.506) cordial usage between female PROs (20.0%) and AMAs (37.5%). There was also no significant difference amongst male competitors in daily servings of: coffee PRO 3.0, AMA 2.2, (χ^2^ (5) 4.69, *p* = 0.455); tea PRO 0.0, AMA 0.4, (χ^2^ (5) 2.42, *p* = 0.788), herbal tea PRO 0.8, AMA 0.6, (χ^2^ (5) 8.354, *p* = 0.138); sugar free carbonated energy drinks PRO 0.1, AMA 0.3, (χ^2^ (2) 1.34, *p* = 0.513); or sugar free carbonated beverages PRO 0.5, AMA 0.6, (χ^2^ (6) 3.82, *p* = 0.701). Amongst females there was no significant difference in daily servings of: coffee PRO 2.0, AMA 2.1, (χ^2^ (5) 3.11, *p* = 0.683); tea PRO 1.0, AMA 0.5, (χ^2^ (5) 2.42, *p* = 0.627); herbal tea PRO 2.0, AMA 1.2, (χ^2^ (5) 8.354, *p* = 0.382); sugar free carbonated energy drinks PRO 1.1, AMA 0.3, (χ^2^ (2) 1.34, *p* = 0.231); or sugar free carbonated beverages PRO 0.2, AMA 0.6, (χ^2^ (6) 3.82, *p* = 0.304). Finally, there was, a non-significant trend (t(29) 1.89, *p* = 0.068) for male PROs to consume more fluids than AMAs (PRO: 5.7 ± 1.3 L, AMA: 4.5 ± 1.5 L). However, there was no significant difference (*z* = 1.09, *p* = 0.273) in fluid intake between female PROs (median = 4.0, IQR 3.5–6 L) and AMAs (median = 3.5, IQR 3–4 L).

## Discussion

To our knowledge, this is the first cross-sectional comparison between AMA and elite PRO natural bodybuilders. Additionally, it is the first which includes and compares male and female, PROs and AMAs. We aimed to determine if: dietary factors, total amount and rate of weight lost, total energy, macronutrient distribution, food item selection, BMR, EED, and supplement usage could distinguish between PROs and AMAs, and if differences were sex-specific. We identified several significant differences in CHO and energy intake between PRO and AMA men, and CHO intake relative to body mass which resulted in a lower EED in male PROs compared to AMAs. Finally, supplement intake was significantly higher in female PROs compared to AMAs.

Male PROs had competed significantly longer than AMAs, and on average, trained for a longer period of time for bodybuilding than AMAs, although this was non-significant (PRO: 17.0 +/− 8.4; AMA 11.2 +/− 6.8 years; *p* = 0.08). Additionally, PRO men weighed more at the start and end than AMA men, but lost a similar amount of body mass, both in total kilograms (PRO: 13.4 +/− 6.1; AMA 13.1 +/− 5.2 kg; *p* = 0.92) and as a percentage of body mass (PRO: 13.8 +/− 5.5; AMA 14.7 +/− 5.4%; *p* = 0.68). Essentially, PRO and AMA male bodybuilders do not differ in their total weight loss. However, PRO men lost a significantly smaller percentage of their body mass per week, and dieted for a greater number of weeks on average than AMA men, although this was non-significant as well (PRO: 28.1 +/− 8.1; AMA 21.0 +/− 9.4 weeks; *p* = 0.06). Overall, it seems that elite PRO men are heavier, and thus, presumably more muscular on average than AMA men (End BMI, PRO: 26.2 +/− 1.0 kg/m2, AMA: 24.0 +/− 1.7 kg/m2 *p* < 0.01), which may be due - at least in part - to a longer period of time spent training as competitive bodybuilders, and possibly a longer history of resistance training overall. This observation is consistent with our previous research which indicated successful bodybuilders have more resistance training and bodybuilding experience than their less successful peers [13]. Additionally, PRO men lose significantly less weight as a proportion of their body mass on average per week, which may be facilitated by longer diets, although future research with larger sample sizes are needed to confirm this finding.

The speculation that PRO men diet longer on average, is partially supported by the significant differences observed between PRO and AMA men in total energy intake. Specifically, PRO men consumed significantly (*p* < 0.01) more energy than AMA men in an absolute sense; however, when energy intake was expressed relative to body mass, this difference was no longer significant (*p* = 0.12). Perhaps more relevant to practice, is that PRO men had a significantly lower EED at the start (PRO: 2.0 +/− 5.5 kcal, AMA: − 3.4 +/− 5.2 kcal *p* = 0.03) and end (PRO -1.1 +/− 6.0 kcal, End AMA: ES -9.3 +/− 6.1 kcal *p* < 0.01) of the preparation period compared to AMA men. Energy availability - an athlete’s energy intake after exercise activity expenditure, relative to LBM – is likely important for bodybuilding contest preparation. For example, athletes expressing chronic low EA experience negative effects on both performance and health [36]; a lower EED among PRO men may therefore reflect an optimised preparation process, in which LBM is better preserved. Indeed, in a recent review of male natural bodybuilding case studies by Fagerberg, a speculative link between low EA and greater losses of muscle mass was proposed [16]. Moreover, Fagerberg [16] speculated that bodybuilders were more likely to suffer from psychological distress associated with chronic low EA which likely has consequences for dietary adherence and general feelings of wellbeing.

In a 2014 review outlining best practices for natural bodybuilding contest preparation [11], a rate of weight loss between 0.5–1% of body mass per week was advised to attenuate losses of LBM. However, in a male natural bodybuilding case study where a rate of weight loss closer to 1% (0.98% of initial body weight/wk) was followed for a shorter time period (13 weeks), 5 kg or 6.7% of total lean mass was lost (42.7% of total body mass lost was lean mass), and the athlete began his diet with an estimated EA of 21 and finished with 13 kcal/kg/LBM [17]. In contrast, the smallest loss of lean mass observed among male natural bodybuilding case studies to date was reported by Rossow et al. [5], in which a rate of weight loss closer to 0.5% (0.52% of initial body weight/wk). This weight loss was undertaken over a longer time period (26 wks), and the athlete lost 2.8 kg or 3.2% of total lean mass (20.1% of total body mass lost was lean mass), and the athlete began his diet with an estimated EA of 25 and finished with 22 kcal/kg/LBM [16]. Therefore, while causative links cannot yet be made, it is possible that even within the recommended best practice weight loss guidelines of 0.5–1% of BW/wk. [11], a loss rate closer to the lower end of this spectrum (facilitated by a longer diet) could possibly result in higher EA. This higher EA may, subsequently ameliorating the symptoms of low EA [35], and possibly preserve more LBM [16]. While more research is needed, slower rates of weight loss per week, longer diets, and subsequently greater EA could possibly be distinguishing, beneficial tactics which separate the practices of elite PRO and AMA male natural bodybuilders.

In addition to the time course and total energy of the diet, macronutrient content differed between PRO and AMA men. Specifically, PRO men reported significantly more total grams of CHO than AMA men, and this difference remained significant when expressed relative to body mass. Also, the non-significantly higher fibre intake (*p* = 0.07) consumed by male PROs likely reflected a higher intake of CHO-dominant foods, which tend to be higher in fibre. It also seems plausible that a higher fibre intake contributes towards a greater satiating effect of the diet, than the lower fibre intake, thereby promoting greater dietary adherence amongst competitors [37]. In our previous comparison between top five placing AMA competitors and non-placing AMA competitors at the BNBF finals, we observed significantly higher CHO intakes among those who placed [13]. The present findings that elite PRO men also consume more energy in the form of a higher CHO diet than AMA men seems to confirm a persistent difference between male British bodybuilders at higher compared to lower competitive levels. Whether this is reflective of best practice, physiological characteristics of those better suited to bodybuilding success (greater glycogen storage capacity, insulin sensitivity in muscle, fuel usage during exercise, metabolic or thermic response to CHO, nutrient partitioning, etc). Coaches and bodybuilders should be caution when it comes to interpreting these findings, bodybuilding is a subjective sport and success is likely dependent on multiple factors beyond CHO intake. Differences in CHO and subsequent energy intake may also simply reflect regional trends among more experienced competitors. For example, energy intakes amongst North and South American bodybuilders reported in the literature are typically lower (range 23 to 46 kcal per kg BW, versus 36 kcal per kg BW in the present investigation) [5–9, 13, 26–28, 31]. Differences also may exists between those competing in the men’s physique and bodybuilding categories, with British bodybuilders seemingly having a tendency to consume more total energy [27, 31]. These differences in energy intakes are important given the consequences for LBM loss, hormonal imbalances, psychological problems and cardiovascular health where EA is chronically less than 25 kcal/kg, as outlined in the aforementioned review [16]. Speculative links between greater energy intake and superior retention of resistance training performance and LBM among energy restricted athletes are also noted in best practice nutrition guidelines for natural bodybuilders [11]. Likewise, a significant correlation (*r* = 0.725; *p* < 0.05) between insulin levels (which would presumably be higher when consuming more CHO) and LBM retention was observed in a cohort of 14 male natural bodybuilders during the final 11 weeks preceding competition [25]. Causative links, however, cannot be inferred from the present design, or from existing research. Rather, these observational differences and associations warrant controlled investigations into whether higher CHO diets can facilitate superior bodybuilding-specific performance. However, it is worth noting that previous CHO recommendations for bodybuilding, of between 4 to 7 g/kg BW, are being utilized by bodybuilders during contest preparation [23].

Where protein and fat is concerned, there was no difference in reported intake between PRO and AMAs between male and female competitors. Although protein and fat declined during preparation in both male and female PROs and AMAs, there was a trend (*p* = 0.06) for protein intake relative to body mass among males. Furthermore, although we did not measure LBM it seems likely competitors were consuming enough protein to meet to bodybuilding recommendations of 2.3–3.3 g/kg LBM for the preservation of muscle in a calorie deficit [11]. The low fat intakes observed in male and female (0.6 to 0.8 g/kg BW) competitors is consistent with other cross sectional studies of bodybuilders [13, 24, 27] and case reports [5–8, 26, 28]. This low fat diet adapted by competitors (13.3 to 22.5% of energy from fat) reflects the low end of the 15 to 30% of total energy recommendations for fat intake proposed for bodybuilding [11]. Interestingly, 55% of male, and 93% of female competitors reported consuming omega 3 fatty acid supplements, presumably to ensure adequate supply of the essential fatty acids eicosapentaenoic acid and docosahexaenoic acid. More research is needed to assess if such low fat intakes are detrimental to bodybuilding performance, particularly when omega-3 fatty acids are consumed.

Importantly, most significant differences in the present study were between the male PROs and AMAs. While few significant differences were observed between PRO and AMA females, it is worth noting that the principal findings observed among males - significantly greater energy and CHO intakes in PROs - also produced the lowest *p* values among women for energy and macronutrient related analyses (*p* = 0.17 to 0.25). While speculative, we propose our female data set may have been underpowered, and thus, similar differences in energy and CHO between AMA and elite PRO men may possibly be present among AMA and elite PRO women, although additional research is required to confirm this speculation. Despite this lack of power, there is a paucity of data available for female physique competitors, and these findings provide initial insights into their dietary practices. One interesting finding is that PRO and AMA competitors consumed different food items from one another and that female competitors may use more artificial sweeteners (*p* = 0.053). It is possible that AMA competitors had not yet established a consistent bodybuilding “nutritional lifestyle” and sought to compensate for this relatively new stress of perceived deprivation during the diet [38] via added non-caloric sweeteners. Interestingly, despite bodybuilding lore that artificial sweeteners may increase body fat [39], male competitors did not exclude them, sugar free cordials or artificially sweetened carbonated beverages, consistent with previous findings [13].

Supplement usage reflected previous reports of British natural bodybuilders [13]. Female PROs used significantly (*p* = 0.03) more supplements than AMA women, which was reflected (although non-significantly; *p* = 0.10) in PRO compared to AMA men as well. While its possible supplement usage influences competitive outcomes, it seems unlikely as most supplements with a proven and relevant ergogenic effect [11] were consumed by both AMAs and PROs. Rather, it is the anecdotal observation of the authors that PRO competitors are more often provided sponsorships for free supplements by supplement companies. Thus, it is possible these differences between PROs and AMAs may be reflective of free supplement access and convenience. Finally, caffeine usage relative to body mass increased in both sexes over time. Although no statistical test was carried out comparing males to females because of the differences between bodybuilding categories, caffeine intake appeared to be higher among females which may reflect the athletes’ smaller size and their tendencies to consume more fat burners, which are typically high in caffeine.

### Limitations

Bodybuilders are known for their strict adherence to bodybuilding menus during contest preparation. Following the same dietary plan for consecutive weeks is common practice in bodybuilding, underreporting however is common in the study of habitual dietary intake. The extent of under report in bodybuilding is unknown, however a recent review reported a 19% difference between double labelled water and energy intake from food records amongst athletic populations [40]. Furthermore, we only obtained a snapshot of participant’s diet from three arbitrary time points (start, middle and end) in the competition preparation. We were therefore unable to capture any additional dietary changes that may have occurred, or practices such as cheat meals, or refeeding. Moreover, participant’s weight and height was self-reported and any inaccuracy in these measurements will have influenced predicted energy requirements as well as energy intake scaled for body mass. It is however worth noting that bodybuilders compete in weight class dependent divisions, and for the purpose of weight loss likely weighing themselves regularly. Furthermore comparisons with athletic populations between self-reported and actual weight and height have noted differences between 0.9 kg and 0.04 cm with the method generally accepted as precise [41]. Moreover, energy intake scaled for bodyweight was broadly similar to the previously published work in British natural bodybuilders corroborating our findings [13]. Furthermore, because of the nature of the study we did not report participant’s fat mass or LBM, which would have helped differentiate between PROs and AMAs. Although it is not unreasonable to assume that the PROs (who obtain this status in this study from being successful at national and international competition) were more muscular and at the start and end of their competition preparation based on BMI. Finally, although we recruited individuals from qualifying events and the PRO grand prix, we did not assess competitive schedule of the participants, which may have influenced the amount of time spent in the offseason, or preparation phase for both the PROs and AMAs.

## Conclusions

There are significant differences among male AMA and elite PROs bodybuilders in years spent competing, body mass, proportion of body mass lost per week, total energy intake, EED, total energy, CHO intake, and relative CHO intake. Furthermore, effect size testing indicated differences between EED and CHO intake between AMA and elite PRO men supporting a practical effect of the aforementioned variables between PROs and AMAs. These differences in nutritional practice may be explained by a combination of higher levels of body mass (presumably LBM) among PROs, less aggressive energy deficits due to higher energy intakes primarily driven by greater CHO consumption, and diets that last longer, which result in similar reductions in body mass, with smaller relative losses per week. Although it should be noted that we did not measure LBM or FM directly. Similar non-significant findings were reported among females, and additional research with larger samples is needed to discern sex differences between female AMAs and PROs. In aggregate, whether these differences reflect best practice, inherent physiological differences between PRO elite competitors and AMAs, or some combination is unknown. However, certain aspects of our findings such as facilitating greater EA and superior body composition outcomes via slower weight loss and longer diets are corroborated in other sports science research disciplines [36, 42]. Thus, for bodybuilding goals we tentatively suggest that longer diets with rates of weight loss closer to 0.5% rather than 1.0% of BW per week, particularly as the athlete gets leaner may be beneficial. Furthermore, so long as this rate of weight loss is sustained, the recommendation that athletes should maintain an EA greater than 25 kcal/kg of LBM to preclude muscle loss and health, seems reasonable [16]. Finally, we encourage future experimental research to explore these avenues for enhancing bodybuilding performance.

## Additional files

Additional file 1: Dietary Assessment of a Natural Bodybuilding Population Questionnaire Version 6.0. (PDF 201 kb)

Additional file 2: **Table S1**. Estimated energy requirements and deficit of competitors with and without the addition of PAL (BMR x 1.7). *p* value, difference in means between PRO and AMA. Data analysed using an Independent T-Test. Statistical significance assumed where *p* < 0.05. Abbreviations: BMR basal metabolic rate, PAL physical activity and lifestyle factor, EED estimated energy deficit, PRO professional, AMA amateur, SD standard deviation. (DOCX 18 kb)

## Data Availability

Please contact author for data requests.

## Abbreviations

AMA: Amateur
BCAA: Branch chain amino acid
BMI: Body mass index
BMR: Basal Metabolic Rate
BNBF: British Natural Bodybuilding Federation
BW: Bodyweight
CHO: Carbohydrate
DFAC: Drug Free Athletes Coalition
EA: Energy availability
EED: Estimated energy deficit
FFM: Fat free mass
LBM: Lean body mass
PAL: Physical activity and lifestyle
PRO: Professional
WADA: World Anti-Doping Agency
WNBF: World Natural Bodybuilding Federation
