# Supplementary material for: Nutritional strategies of British professional and amateur natural bodybuilders during competition preparation
Source: J Int Soc Sports Nutr. 2019 Aug 22;16:35. doi: 10.1186/s12970-019-0302-y (PMC6704518; doi:10.1186/s12970-019-0302-y)
Supplement: Supplementary file 1 — Dietary Assessment of a Natural Bodybuilding Population Questionnaire Version 6.0. (PDF 201 kb) [file 12970_2019_302_MOESM1_ESM.pdf]

Dietary Assessment of a Natural Bodybuilding Population

Sheffield Hallam University

Food and Nutrition Group

Dr. Andrew Chappell

Questionnaire Number:

Version 6.0

PLEASE ANSWER All QUESTIONS RELATED TO YOUR CONTEST PREPARATION

If you are unsure about any of the questions then please ask a research for clarification

Competitor Information

Competitor Number:___________ Competitor Class:____________ Age:__________________

Smoking Status (please circle): Smoker Non Smoker Smoke During Prep

Year's bodybuilding training:______ Year's competing:_______ Competitions this season: ____

Weeks dieting for this competition (including qualifier if applicable):______________________

Current contest weight (kg):________________ Weight at start of preparation (kg):__________

Height (cm):____________ Bodyfat % and the method used to estimate:__________________

____

Highest bodybuilding accolade (example: BNBF British Men's open lightweight 3rd 2013):

____

Fluid Intake

Typical daily fluid intake (litres):_____________

Do you use artificial sweeteners? Yes/No Do you use sugar free cordials/squash? Yes/No

Beverage intake during contest preparation, please tick (✓) as appropriate

**Standard Serving: Can of pop 330ml, Cup of tea 150ml, take away coffee 250ml**									
Normal serving	Less than once a month	1 - 3 per month	1 per week	2 - 4 per week	5 - 6 per week	1 per day	2 - 3 per day	4 - 5 per day	6+ per day
Coffee									
Espresso									
Tea									
Herbal tea									
Energy drink									
Fizzy drinks									
Diet fizzy drinks									
Alcohol									

Supplementation

Supplement intake during contest preparation, please tick (✓) as appropriate

**Supplement**	**please tick (✓)**	**Brand**
Multivitamin		
Vitamin C		
Vitamin D		
Mineral supplement		
Joint supplement		
Omega-3/Cod liver oil		
Protein powder		
BCAA		
Individual amino acid		
Carbohydrate supplement		
Creatine (directly or indirectly)		
Fat burners		
Pre-workout supplements		
Protein/Flapjack Bars		
Other		

Dietary Approach

Which of the following best describes your dietary approach? Circle where appropriate:

**Diet Style:**	Clean eating	If it fits your macro's	Cheat meals
**High:**	Protein	Carbohydrate	Fat
**Medium:**	Protein	Carbohydrate	Fat
**Low:**	Protein	Carbohydrate	Fat

Competition Diet

In the space below, please record the diet followed (in either portion sizes or grams) at the start, middle and end (not including peak week) of your contest preparation.

Initial Diet (first few weeks)	Middle Diet (halfway phase)	End Diet (final weeks)
**Example Diet**<br>***M1:****100g of Weatabix in water, banana, 3 whole boiled eggs, 40g of whey (Extreme Nutrition)*<br>***M2:*** *1 CNP pro flapjack (orange flavour), small apple 70g*<br>***M3:*** *150g of steamed cod,40g of spinach, 30g of green olives, 200g of boiled white potatoes*<br>***M4:*** *Repeat meal 3*<br>***M5:*** *Repeat meal 1*<br>***Pre Workout:*** *30g NO Explode Lime flavour (BSN)*<br>***Post Exercise:*** *80g Build and Recover (Extreme Nutrition)*<br>***M7:****70g Low Fat Cottage Cheese (Tesco), 35g Pro Peptide (CNP)*<br>***Drinks:*** *3 Cups of instant black coffee with meals (Kenco)*<br>*1 Can of Pepsi Max*	**Example Diet**<br>***M1:****50g of Weatabix in water, banana, 3 whole boiled eggs.40g of whey (Extreme Nutrition)*<br>***M2:*** *1 CNP pro flapjack (orange flavour), small apple 70g*<br>***M3:*** *120g of steamed cod,40g of spinach, 30g of green olives, 150g of boiled white potatoes*<br>***M4:*** *Repeat meal 3*<br>***M5:*** *Repeat meal 1*<br>***Pre Workout:*** *30g NO Explode Lime flavour (BSN)*<br>***Post Exercise:*** *60g Build and Recover (Extreme Nutrition)*<br>***M7:****60g Low Fat Cottage Cheese (Tesco), 35g Pro Peptide (CNP)*<br>***Drinks:*** *2 Cups of instant black coffee with meals (Kenco)*<br>*2 Cans of Pepsi Max*	**Example Diet**<br>***M1:*** *60g of banana and 6 whole boiled eggs, 50g of whey (Extreme Nutrition)*<br>***M2:*** *1 CNP pro flapjack (orange flavour), small apple 70g*<br>***M3:*** *120g of steamed cod,40g of spinach, 30g of green olives, 100g of boiled white potatoes*<br>***M4:*** *Repeat meal 3*<br>***M5:*** *Repeat meal 1*<br>***Pre Workout:*** *30g NO Explode Lime flavour (BSN)*<br>***Post Exercise:*** *50g Build and Recover (Extreme Nutrition)*<br>***M7:****50g Low Fat Cottage Cheese (Tesco), 50g Pro Peptide (CNP)5g*<br>***Drinks:*** *6 Cups of instant black coffee with meals (Kenco)*<br>*3 Cans of Pepsi Max*
		**(more space available on the next page)**

Resistance Training Programme

Please circle as appropriate

Resistance training at the Start of contest preparation (first few weeks)

Number of resistance training sessions per week:		1	2	3	4	5	6	7+
Number of chest, sessions per week:		1	2	3	4	5	6	7+
Number of back training sessions per week:		1	2	3	4	5	6	7+
Number of quadriceps training sessions per week:		1	2	3	4	5	6	7+
Number of exercises for chest, back or quads:		1	2	3	4	5	6	7+
Typical sets per exercise for chest, back or quads:		1	2	3	4	5	6	7+
Number of sets between 1 - 5 repetitions:	0	1	2	3	4	5	6	7+
Number of sets above 13 repetitions:	0	1	2	3	4	5	6	7+

Resistance training in the Middle of contest preparation (halfway phase)

Number of resistance training sessions per week:		1	2	3	4	5	6	7+
Number of chest, sessions per week:		1	2	3	4	5	6	7+
Number of back training sessions per week:		1	2	3	4	5	6	7+
Number of quadriceps training sessions per week:		1	2	3	4	5	6	7+
Number of exercises for chest, back or quads:		1	2	3	4	5	6	7+
Typical sets per exercise for chest, back or quads:		1	2	3	4	5	6	7+
Number of sets between 1 - 5 repetitions:	0	1	2	3	4	5	6	7+
Number of sets above 13 repetitions:	0	1	2	3	4	5	6	7+

Resistance training at the End of contest preparation (final weeks)

Number of resistance training sessions per week:		1	2	3	4	5	6	7+
Number of chest, sessions per week:		1	2	3	4	5	6	7+
Number of back training sessions per week:		1	2	3	4	5	6	7+
Number of quadriceps training sessions per week:		1	2	3	4	5	6	7+
Number of exercises for chest, back or quads:		1	2	3	4	5	6	7+
Typical sets per exercise for chest, back or quads:		1	2	3	4	5	6	7+
Number of sets between 1 - 5 repetitions:	0	1	2	3	4	5	6	7+
Number of sets above 13 repetitions:	0	1	2	3	4	5	6	7+

Cardiovascular Training Programme

Start of contest preparation (first few weeks)

Did you engage in fasted cardio? Yes/ No

Number of high intensity interval training (HIIT) or steady state cardio performed per week?

HIIT: 0 1 2 3 4 5 6 7 8 Other: ___________

Duration and any additional information: ____

Stead State: 0 1 2 3 4 5 6 7 8 Other: ___________

Duration and any additional information: ____

Habitual cardio sessions, e.g walking to and from work, occupational cardio etc.

Number of sessions: 0 1 2 3 4 5 6 7 8 Other: _____

Duration and additional information: ____

Middle of contest preparation (halfway phase)

Did you engage in fasted cardio? Yes/ No

Number of high intensity interval training (HIIT) or steady state cardio performed per week?

HIIT: 0 1 2 3 4 5 6 7 8 Other: ___________

Duration and any additional information: ____

Stead State: 0 1 2 3 4 5 6 7 8 Other: ___________

Duration and any additional information: ____

Habitual cardio sessions, e.g walking to and from work, occupational cardio etc.

Number of sessions: 0 1 2 3 4 5 6 7 8 Other: _____

Duration and additional information: ____

End of contest preparation (final weeks)

Did you engage in fasted cardio? Yes/ No

Number of high intensity interval training (HIIT) or steady state cardio performed per week?

HIIT: 0 1 2 3 4 5 6 7 8 Other: ___________

Duration and any additional information:____

Stead State: 0 1 2 3 4 5 6 7 8 Other: ___________

Duration and any additional information:____

Habitual cardio sessions, e.g walking to and from work, occupational cardio etc.

Number of sessions: 0 1 2 3 4 5 6 7 8 Other: _____

Duration and additional information: ____

End of Questionnaire

[Blank Page]
