# Supplementary material for: Nutritional strategies of British professional and amateur natural bodybuilders during competition preparation
Source: J Int Soc Sports Nutr. 2019 Aug 22;16:35. doi: 10.1186/s12970-019-0302-y (PMC6704518; doi:10.1186/s12970-019-0302-y)
Supplement: Supplementary file 2 — Table S1. Estimated energy requirements and deficit of competitors with and without the addition of PAL (BMR x 1.7). p value, difference in means between PRO and AMA. Data analysed using an Independent T-Test. Statistical significance assumed where p < 0.05. Abbreviations: BMR basal metabolic rate, PAL physical activity and lifestyle factor, EED estimated energy deficit, PRO professional, AMA amateur, SD standard deviation. (DOCX 18 kb) [file 12970_2019_302_MOESM2_ESM.docx]

**Table S1. Estimated energy expenditure and deficit of British natural bodybuilders during competition preparation.**

|  | **Male** | | | | |  | **Female** | | | | |  | **Male** | | **Female** | |
| --- | --- | --- | --- | --- | --- | --- | --- | --- | --- | --- | --- | --- | --- | --- | --- | --- |
| **Start** | **PRO** | **SD** | **AMA** | **SD** | ***p* value** |  | **PRO** | **SD** | **AMA** | **SD** | ***p* value** |  | **Mean** | **SD** | **Mean** | **SD** |
| **BMR** | 1973 | 96 | 1958 | 102 | 0.712 |  | 1390 | 49 | 1399 | 56 | 0.754 |  | 1961 | 100 | 1396 | 52 |
| **PAL** | 3353 | 163 | 3195 | 687 | 0.296 |  | 2362 | 83 | 2378 | 96 | 0.754 |  | 3233 | 604 | 2373 | 89 |
| **End** |  |  |  |  |  |  |  |  |  |  |  |  |  |  |  |  |
| **BMR** | 1819 | 76 | 1783 | 78 | 0.267 |  | 1317 | 40 | 1312 | 40 | 0.827 |  | 1792 | 78 | 1314 | 38 |
| **PAL** | 3092 | 129 | 3031 | 133 | 0.269 |  | 2239 | 64 | 2231 | 68 | 0.827 |  | 3046 | 132 | 2334 | 64 |
| **Estimated Energy Deficit** | | | |  |  |  |  |  |  |  |  |  |  |  |  |  |
| **Start** | **PRO** | **SD** | **AMA** | **SD** | ***p* value** |  | **PRO** | **SD** | **AMA** | **SD** | ***p* value** |  | **Mean** | **SD** | **Mean** | **SD** |
| **BMR** | 1560.5 | 524.9 | 1089.3 | 466.1 | 0.088 |  | 1073.4 | 538.6 | 900.6 | 389.9 | 0.549 |  | 1203.5 | 515.0 | 962.3 | 436.1 |
| **PAL** | 179.7 | 532.5 | -226.2 | 629.6 | 0.034 |  | 100.6 | 551.8 | -78.6 | 421.8 | 0.550 |  | -127.8 | 624.9 | -14.6 | 459.4 |
| **End** |  |  |  |  |  |  |  |  |  |  |  |  |  |  |  |  |
| **BMR** | 1199.4 | 516.6 | 546.6 | 447.4 | 0.008 |  | 518.7 | 269.9 | 348.5 | 432.0 | 0.383 |  | 704.9 | 537.8 | 409.2 | 380.0 |
| **PAL** | -73.9 | 485.1 | -701.5 | 462.7 | 0.008 |  | -403.3 | 248.5 | -570.2 | 445.4 | 0.386 |  | -549.4 | 535.4 | -510.6 | 384.6 |

*Table S1. Estimated energy requirements and deficit of competitors with and without the addition of PAL (BMR x 1.7). p value, difference in means between PRO and AMA. Data analysed using an Independent T-Test. Statistical significance assumed where p < 0.05*

*Abbreviations: BMR basal metabolic rate, PAL physical activity and lifestyle factor, EED estimated energy deficit, PRO professional, AMA amateur, SD standard deviation*
